# Prevalence and characterization of *Salmonella enterica* from the feces of cattle, poultry, swine and hedgehogs in Burkina Faso and their comparison to human *Salmonella* isolates

**DOI:** 10.1186/1471-2180-13-253

**Published:** 2013-11-11

**Authors:** Assèta Kagambèga, Taru Lienemann, Laura Aulu, Alfred S Traoré, Nicolas Barro, Anja Siitonen, Kaisa Haukka

**Affiliations:** 1 Bacteriology Unit, Department of Infectious Disease Surveillance and Control, National Institute for Health and Welfare (THL), Helsinki, Finland; 2 Laboratoire de Biologie Moléculaire et d’Epidémiologie et de Surveillance Bactéries et Virus transmis par les Aliments, CRSBAN, Département de Biochimie-Microbiologie, UFR-SVT/Université de Ouagadougou, Ouagadougou, Burkina Faso; 3 Department of Food and Environmental Sciences, Division of Microbiology, University of Helsinki, Helsinki, Finland

**Keywords:** *Salmonella*, Serotypes, Antimicrobial resistance, Genetic relatedness, PFGE

## Abstract

**Background:**

Production and wild animals are major sources of human salmonellosis and animals raised for food also play an important role in transmission of antimicrobial resistant *Salmonella* strains to humans. Furthermore, in sub-Saharan Africa non-typhoidal *Salmonella* serotypes are common bloodstream isolates in febrile patients. Yet, little is known about the environmental reservoirs and predominant modes of transmission of these pathogens. The purpose of this study was to discover potential sources and distribution vehicles of *Salmonella* by isolating strains from apparently healthy slaughtered food animals and wild hedgehogs and by determining the genetic relatedness between the strains and human isolates. For this purpose, 729 feces samples from apparently healthy slaughtered cattle (n = 304), poultry (n = 350), swine (n = 50) and hedgehogs (n = 25) were examined for the presence of *Salmonella enterica* in Burkina Faso. The isolates were characterized by serotyping, antimicrobial-susceptibility testing, phage typing, and pulsed-field gel electrophoresis (PFGE) with *Xba*I and *Bln*I restriction enzymes.

**Results:**

Of the 729 feces samples, 383 (53%) contained *Salmonella*, representing a total of 81 different serotypes. *Salmonella* was present in 52% of the cattle, 55% of the poultry, 16% of the swine and 96% of the hedgehog feces samples. Antimicrobial resistance was detected in 14% of the isolates. *S.* Typhimurium isolates from poultry and humans (obtained from a previous study) were multiresistant to the same antimicrobials (ampicillin, chloramphenicol, streptomycin, sulfonamides and trimethoprim), had the same phage type DT 56 and were closely related in PFGE. *S.* Muenster isolates from hedgehogs had similar PFGE patterns as the domestic animals.

**Conclusions:**

Based on our results it seems that production and wild animals can share the same *Salmonella* serotypes and potentially transmit some of them to humans. As the humans and animals often live in close vicinity in Africa and the hygiene control of the meat retail chain is defective, high *Salmonella* carriage rates of the animals can pose a major public health risk in Burkina Faso. This underlines the necessity for a joint and coordinated surveillance and monitoring programs for salmonellosis in Africa.

## Background

*Salmonella* is one of the major zoonotic foodborne pathogens worldwide. It can cause a variety of clinical manifestations from mild gastroenteritis to bacteremia and typhoid fever. The global burden of nontyphoidal *Salmonella* gastroenteritis has been estimated to be 93.8 million cases of gastroenteritis each year, with 155 000 deaths [1]. In Africa, non-typhoidal *Salmonella* has consistently been reported as a leading cause of bacteremia among immuno-compromised people, infants and newborns [2,3]. However, the sources and transmission routes of *Salmonella* in developing countries are poorly understood due to the lack of coordinated national epidemiological surveillance systems [4,5]. In general, the primary sources of salmonellosis are considered to be food-producing animals such as cattle, poultry and swine [6]. The pathogens are mainly disseminated by trade in animals and uncooked animal food products [7]. The process of removing the gastrointestinal tract during slaughtering of food animals is regarded as one of the most important sources of carcass and organ contamination with *Salmonella* at abattoirs [8]. Also asymptomatic pet animals are a potential source of infection, especially species with high fecal carriage rates of *Salmonella*[9]. African pygmy hedgehogs kept as pets have previously been associated with cases of human salmonellosis [10]. The development and the accumulation of resistance to antimicrobials in foodborne pathogens are a major problem for public health. Multi-resistant *Salmonella* may acquire their resistance genes from microbiota of production animals before being transmitted to humans through food chain [11,12].

Due to the lacking surveillance programs in Burkina Faso, as in the most of Africa, information on the prevalence of *Salmonella* and other enteropathogens in food stuffs is limited. However, our previous study on the prevalence of enteric bacteria on retail meats sold at the markets in Ouagadougou, Burkina Faso, revealed that 37% of the chicken, 13% of the beef intestines, and 7% of the mutton samples were contaminated by *Salmonella*[13]. The most common serotypes detected were *S.* Derby and *S.* Tilene. In a following broader study on chicken carcasses in Burkina Faso, up to 57% of the carcasses were found to be contaminated by *Salmonella*, *S.* Derby again being the most common serotype [14]. In order to better understand the origin of the pathogens, in the current study, we sampled the feces of the common food animals during slaughter. Since previously *S.* Tilene has mainly been recovered from African pigmy hedgehogs kept as pets in North America or Europe [15,16], we included hedgehogs, which are common on the grassy pastures in Burkina Faso and also consumed as food, in our study.

The specific aims of our study were: first, to estimate the prevalence of *Salmonella* in the feces of slaughtered cattle, poultry and swine, as well as in the feces of hedgehogs in Burkina Faso; second, to identify the serotype of *Salmonella* isolates; third, to determine the sensitivity of the isolates to the antimicrobial agents; and finally, to assess the genetic relatedness of the isolates from the feces of the animals and from the local children using pulsed-field gel electrophoresis (PFGE).

## Results

### *Salmonella* prevalence and the serotypes

**Figure 1:**
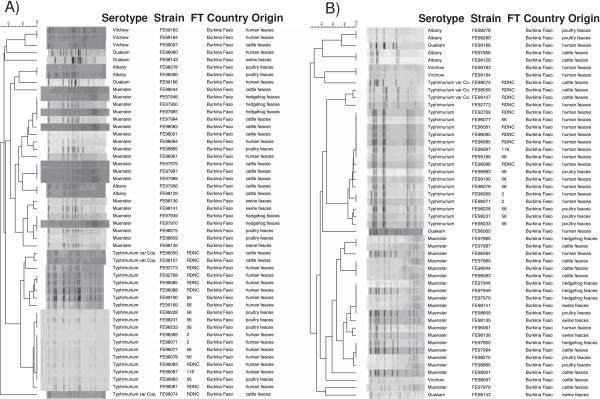
**Pulsed-field gel analysis with *Xba*I (A) and *Bln*I (B) to assess the genetic similarity of the *Salmonella *isolates from animal and human feces from Burkina Faso.** Fifty *Salmonella* strains belonging to serotypes Muenster (n = 20), Typhimurium (n = 17), Typhimurium var. Copenhagen (n = 3), Albany (n = 4), Virchow (n = 3) and Ouakam (n = 3) were analysed. FT = phage type.

*Salmonella* was isolated from 383 (53%) of the total of 729 feces samples from apparently healthy animals. Isolates were obtained from 159 (52%) of the cattle feces, 192 (55%) of the chicken feces, 8 (16%) of the swine feces and 24 (96%) of the hedgehog feces (Table 1). Of the 383 isolates, 382 belonged to *S. enterica* ssp. *enterica* and one was S. *enterica* ssp. *salamae*. 364 of the *S. enterica* ssp. *enterica* isolates could be serotyped in detail, while for 18 isolates only the *Salmonella* group could be assigned. 60 different serotypes were found from the cattle, 41 from the chicken, 5 from the swine and 8 from the hedgehog feces. The predominant serotypes were *S*. Drac and *S.* Muenster in the cattle, *S*. Derby and *S.* Chester in the poultry and *S*. Muenster in both the swine and hedgehog feces. The 3 *S.* Typhimurium isolates from the cattle all belonged to variant Copenhagen. Phage typing divided the *S.* Typhimurium isolates further into three definite phage types: DTs 2, 56 and 116 (Figure 1). In addition, 9 strains were RDNC (reacts but do not conform).

**Table 1:** ***Salmonella enterica *** **serotypes isolated from cattle, poultry, swine and hedgehog feces and their antimicrobial resistance patterns**

***Salmonella *** **serotypes**	**Cattle feces (n = 304)**	**Poultry feces (n = 350)**	**Swine feces (n = 50)**	**Hedgehog feces (n = 25)**	**Total (n = 729)**	**Antimicrobial resistance patterns**	
						**Resistant**^**a**^	**Intermediate**^**a**^
*S*. Abaetetuba	1	1	-	-	2	-	1**P**str-tet, 1**C**str
*S*. Abony	-	1	-	-	1	-	-
*S*. Adelaide	-	1	-	-	1	-	-
*S*. Agona	-	3	-	-	3	-	1**P**str-sul, 1**C**str
*S*. Albany	2	2	-	-	4	-	1**P**tet, 1**C**str
*S*. Anatum	-	1	-	-	1	-	1**P**str
*S*. Ank	-	1	-	4	5	-	4**H**str, 1**P**str
*S*. Antwepen	1	-	-	-	1	-	1**C**str
*S*. Apeyeme	2	3	-	-	5	2**C**str	3**P**str
*S*. Banana	1	2	-	1	4	1**H**str	1**C**str
*S*. Bareilly	1	-	-	-	1	-	1**C**str
*S*. Bargny	1	-	-	-	1	-	1**C**str
*S*. Binningen	-	2	-	-	2	-	-
*S*. Brancaster	1	3	-	-	4	-	1**C**str, 1**P**str, 1**P**str-tet
*S*. Bredeney	5	2	-	-	7	-	4**C**str, 1**P**str
*S*. Brive	1	-	-	-	1	-	1**C**str
*S*. Carmel	1	-	-	-	1	-	-
*S*. Carno	1	-	-	-	1	-	-
*S*. Chandans	2	-	-	-	2	-	2**C**str
*S*. Chester	1	31	-	-	32	1**P**mec	29**P**str, 1**C**str, 1**P**str-tet
*S*. Chomedey	4	-	-	-	4	-	4**C**str
*S*. Colindale	1	-	-	-	1	-	1**C**str
*S*. Colobane	2	-	-	-	2	1**C**str	1**C**str
*S*. Dahra	2	-	-	-	2	-	1**C**str-tet
*S*. Dakar	1	-	-	-	1	1**C**str	-
*S*. Derby	-	51	-	-	51	5**P**tet, 3**P**str, 1**P**chl, 1**P**sul	22**P**str , 1**P**sul, 1**P**sul-tet, 7**P**str-tet, 7**P**str-sul, 2**P**str-sul-tet
*S*. Drac	26	-	-	1	27	4**C**str	1**H**str, 22**C**str
*S*. Duisburg	-	1	-	-	1	-	1**P**str
*S*. Eastbourne	2	2	-	-	4	-	2**C**str, 1**P**str, 1**P**str-tet
*S*. Farakan	3	-	-	-	3	1**C**str	1**C**str
*S*. Freetown	-	1	-	-	1	-	1**P**str
*S*. Fresno	-	4	-	-	4	1**P**str	1**P**str
*S*. Frintrop	1	-	-	-	1	-	1**C**str
*S*. Fufu	1	-	-	-	1	-	1**C**str
*S*. Galiema	-	2	-	-	2	-	2**P**str
*S*. Gokul	1	-	-	-	1		1**C**str
*S*. Hato	5	22	-	-	27	1**P**amp-str-sul-tet-tmp, 1**P**amp, 1**P**str	8**P**str, 1**P**sul-tet, 2**P**str-tet, 1**P**tet, 1**C**str
*S*. Hillingdon	-	1	-	-	1	-	1**P**str
*S*. Ikeja	1	-	-	-	1	-	1**C**str
*S*. Ilala	2	-	1	-	2	-	1**S**str
*S*. Kaapstad	-	4	1	-	5	-	1**P**str, 1**S**str
*S*. Kalamu	1	-	-	-	1	-	-
*S*. Kalina	2	-	-	-	2	-	1**C**str
*S*. Kingston	2	3	-	-	5	-	1**P**str, 1**C**str
*S*. Kokomlemle	2	1	-	-	3	-	1**P**str, 1**C**str
*S*. Korlebu	2	-	-	-	2	2**C**str	-
*S*. Lagos	4	2	-	-	6	2**P**str	1**P**tet, 2**C**str
*S*. Moero	1	-	-	-	1	-	-
*S*. Monschaui	1	1	-	3	5	3**H**str	1**P**str
*S*. Muenster	17	6	3	11	37	1**C**amp, 1**C**str, 1**P**nal, 1**H**sul, 1**H**str	5**H**str, 6**C**str, 4**P**str, 2**S**str, 1**H**tet
*S*. Nima	3	-	-	-	3	-	-
*S*. Nottingham	2	1	-	-	3	-	1**P**str-tet
*S*. Oranienburg	1	-	-	-	1	-	1**C**str
*S*. Othmarschen	1	-	-	-	1	1**C**str	-
*S*. Ouakam	-	-	1	-	1	-	1**S**str
*S*. Poona	2	1	-	-	3	-	1**P**str, 2**C**str
*S*. Rissen	1	-	-	-	1	-	-
*S*. Ruiru	8	-	-	-	8	1**C**str, 1**C**str-tet	3**C**str
*S*. Saintpaul	-	1	-	-	1	-	1**P**tet
*S.* Salford	1	-	-	-	1	-	-
*S*. Schwarzengrund	1	3	-	-	4	-	1**C**str , 3**P**str
*S*. Senftenberg	-	8	-	2	10	-	4**P**str, 2**P**str-tet, 1**P**str-sul-tet
*S*. Shangani	-	1	-	-	1	-	1**P**str -sul
*S*. Soumbedioune	4	-	-	-	4	-	3**C**str
*S*. Stanley	-	-	-	1	1	-	1**H**str
*S*. Stanleyville	-	1	-	-	1	-	1**P**str-tet
S.Tennessee	3	-	-	-	3	-	1**C**str
*S*. Trachau	1	1	-	-	2	1**C**str	1**P**str
*S*. Typhi	-	1	-	-	1	1**P**str	-
*S*. Typhimurium	3	4	-	-	7	4**P**amp-chl-str-sul-tmp, 3**C**str	-
*S*. Umbadah	1	-	-	-	1	-	-
*S*. Umbilo	1	-	-	-	1	-	1**C**str
*S*. Urbana	13	1	2	-	16	1**C**chl-tmp-nal-mec	4**C**str, 1**C**str-ftx, 2**C**str-tet, 1**C**str-cip, 1**P**str, 1**S**str
*S*. Virchow	1	-	-	-	1	-	1**C**str
*S*. Waycross	2	1	-	-	3	1**C**str	1**C**str, 1**P**cip
*S*. Yoruba	1	-	-	-	1	-	1**C**str
*S*. group B 4,5,12:-:-	1		-	-	1	1**C**str-tet	-
*S*. group C 6,7,14:d:-	1	9	-	-	10	-	5**P**str-sul, 4**P**str, 1**C**str
*S*. group E 3,10:e,h:-	1	5	-	-	6	-	1**P**str-sul-tet, 1**P**str, 1**C**str
*S*. group G 13,22:z:-	-	-	-	1	1	-	1**H**str
*Salmonella enterica* ssp. *salamae*	1	-	-	-	1	-	-
**Total**	**159**	**192**	**8**	**24**	**383**	**52**	**247**
	**(52%)**	**(55%)**	**(16%)**	**(96%)**	**(53%)**	**(7%)**	**(34%)**

^a^For example, entry 7**P**str-tet, means that 7 isolates from poultry feces were resistant/intermediate to streptomycin and tetracycline. Abbreviations: **C**, cattle feces; **P**, poultry feces; **S**, swine feces; **H**, hedgehog feces, amp, ampicillin; chl, chloramphenicol; str, streptomycin; sul, sulphonamides; tmp, trimethoprim; tet, tetracycline; nal, nalidixic acid; cip, ciprofloxacin; ftx, cefotaxime; mec, mecillinam.

### Antimicrobial resistance

On the whole, 52 (14%) of the 383 *Salmonella* isolates were resistant to one or more antimicrobials tested: 23 of these were from the cattle, 23 from the poultry and 6 from the hedgehog feces (Table 1). The salmonella isolates from the swine feces were susceptible to the tested antimicrobials. Six isolates were multiresistant: 4 *S*. Typhimurium isolates from the poultry feces (ampicillin, chloramphenicol, streptomycin, sulfonamides and trimethoprim), 1 *S.* Hato isolate from the poultry feces (ampicillin, streptomycin, sulfonamides, tetracycline, trimethoprim) and 1 *S.* Urbana isolate from the cattle feces (chloramphenicol, trimethoprim, nalidixic acid and mecillinam). Out of the 383 isolates, 247 (64%) showed decreased sensitivity (i.e. were intermediate) to one or more antimicrobial, especially to streptomycin, tetracycline and sulphonamides (Table 1). Two isolates (*S.* Urbana and *S.* Waycross) had decreased sensitivity to ciprofloxacin and one (*S.* Urbana) to cefotaxime. The MIC values for the nalidixic acid resistant isolates were 0.023 μg/ml (*S.* Muenster) and 0.032 μg/ml (*S.* Urbana).

### Genetic relatedness by PFGE

To determine the genotypic relatedness of the *Salmonella* isolates recovered from the cattle, poultry, swine and hedgehog feces and to compare them to human isolates from Burkina Faso [17], a total of 50 isolates were subjected to PFGE analysis with *Xba*I and *Bln*I restriction enzymes (Figure 1). Genetic relatedness of the isolates belonging to the same serotype ranged from approximately 70% to 100%. *S.* Typhimurium isolates from the poultry and human feces clustered closely together. *S. *Muenster isolates obtained from the cattle and swine feces were different, but both clustered closely together with some hedgehog isolates (Figure 1). Two *S.* Typhimurium var. Copenhagen isolates from the cattle feces clustered together with the *S.* Typhimurium isolates when *Xba*I was used, whereas all three were distinct from S. Typhimurium when *Bln*I was used. *S.* Albany isolates from the cattle and poultry feces clustered separately using both enzymes.

## Discussion

We detected high prevalence of *Salmonella enterica* ssp. *enterica* in the feces of the production animals slaughtered for human consumption in Burkina Faso. *Salmonella* was especially common in the poultry (55%) and cattle (52%) feces samples. The levels of *Salmonella* in poultry can vary depending on the country, the nature of the production system and the specific control measures in place. In some EU countries chicken flocks are virtually free from *Salmonella* whereas in the US a contamination rate up to 60% was detected [18]. In Japan, *Salmonella* was isolated from 36% of the broiler fecal samples [19]. In Gambia, the detected rate of *Salmonella* in chicken feces was higher, 67% [20], than what we detected from the chicken feces. In comparison, only 11% of chicken reared at intensive poultry farms in Nigeria were found to be infected [21].

The levels of *Salmonella* rates reported in beef are usually lower than in chicken. *Salmonella* carriage was reported to be 1.4% in cattle in Great Britain [22] and 0.5% in Japan [19]. In Ethiopia, 4% of the feces of slaughtered cattle were contaminated by *Salmonella*[23]. The high rate of *Salmonella* detected in our study might be explained partly by the method used for strain isolation and partly by the animal husbandry practices. In Burkina Faso, cows and sheep mostly roam freely at pasture in the bush. The wild animals, such as hedgehogs, living in such places can contaminate grass with their excreta, which, as shown in our study, can have high carriage rate of *Salmonella*. We found 16% of the swine feces samples to be contaminated by *Salmonella. Salmonella* contamination rates for pigs reported in literature vary from 9% to 23% in Europe [18,22,24], to 3% of porcine fecal samples in Japan [19] and 8% in Kenya [25]. In accordance to the high rates of *Salmonella* detected in the feces samples, our previous studies on the prevalence of *Salmonella* in retail meats and beef intestines in Burkina Faso also revealed high numbers of *Salmonella*, especially in chicken (37-57%) [13,14].

Several of the serotypes isolated in this study, including *S*. Typhimurium, *S*. Muenster, *S*. Derby, *S*. Virchow, *S*. Hato, *S*. Bredeney, *S*. Stanley and *S*. Anatum, have frequently been implicated in outbreaks or sporadic cases of human illness [26]. In Africa, as elsewhere in the world, *S*. Enteritidis and *S*. Typhimurium are the most common causes of human salmonellosis [27]. Interestingly, *S*. Enteritidis was not recovered from the animal feces in our study and not from the human isolates in Burkina Faso either [17]. The main serotypes found in both animal and human feces samples from Burkina Faso included *S*. Typhimurium (from poultry) and *S*. Muenster (from all the studied animal species). *S*. Derby was the most common serotype we detected in the chicken feces, as it was in the chicken carcasses [13,14]. World-wide, a wide range of *Salmonella* serotypes have the ability to colonize poultry: *S*. Typhimurium, *S*. Enteritidis, *S*. Hadar, *S*. Virchow, *S*. Infantis and, recently, *S*. Paratyphi B var. Java have all been frequently isolated from poultry in several countries [18], none of which were among the most common serotypes in poultry in Burkina Faso. Elsewhere in Africa, *S*. Enteritidis was the most common serotype detected in chicken feces in Zimbabwe [28] and *S*. Typhimurium in Algeria [29]. Notably, we isolated one *S*. Typhi strain from the chicken feces, as we did previously from a chicken carcass [14].

The *S*. Typhimurium isolates from chicken feces in Burkina Faso were multi-resistant to the commonly available antimicrobials ampicillin, chloramphenicol, streptomycin, sulfonamides and trimethoprim. This is a typical pattern found in the *Salmonella* strains with a sub-Saharan distinct genotype causing epidemic invasive disease [30]. Bacteremia caused by multi-resistant *S*. Typhimurium strains is a serious public health problem in Africa and they are significantly associated with increased mortality [31]. Such *S*. Typhimurium isolates have been reported from e.g. Zaire [31], Kenya [32], Malawi [32] and Central Africa [33]. Although antimicrobial use for animals is under veterinary prescription control in Burkina Faso, farmers still use unprescribed antimicrobials as growth promoters or treatment for cattle, poultry and swine. This practice leads into a possibility that bacterial resistance developing in the food animals transfers to the human population thus posing a risk for public health by spreading of the resistance [34]. It would be essential to study the genotype of our *S*. Typhimurium isolates from poultry further in order to know if the invasive genotype also occurs in animals as the environmental reservoirs and host ranges of invasive salmonella strains in Africa are still unknown [35]. Our *S*. Typhimurium isolates from chicken and humans had the same phage type DT 56. This phage type was in Kenya among the most common phage type from adult patients [36]. In developed countries, a phage type DT 104 has often been associated with outbreaks of multiresistant *S*. Typhimurium infection in both man and animals [37]. Only two isolates in our study was resistant to the newer antimicrobials; S. Muenster from the poultry feces was resistant to nalidixic acid, as was *S*. Urbana from the cattle feces, furthermore, its sensitivity to ciprofloxacin and cefotaxime was decreased.

PFGE provides valuable phylogenetic-relationship inference for *Salmonella* at serotype and strain level [38,39]. Our cluster analysis revealed close genetic relationship between some human and animal strains belonging to the same serotypes. Notable similarity of the chicken and human isolates indicates that chicken may be a major source of *Salmonella* transmission to humans. Also in Senegal, a study detected a high degree of similarity among *S*. Hadar, *S*. Brancaster and *S*. Enteritidis from poultry meat and humans by using PFGE [40]. Besides through food, direct transmission from chicken to humans could easily happen in Burkina Faso, since chickens roam free scattering their feces anywhere in the house yards. Although, in these surroundings it is also possible that it is rather chicken which get transiently infected with the typical human *Salmonella* strains. However, the study conducted recently on isolates from infected children and their households in the Gambia did not support the hypothesis that humans and animals living in close contact in the same household carry genotypically similar *Salmonella* serotypes [20].

We found out that the prevalence of *Salmonella* in hedgehog feces was particularly high (96%). In Burkina Faso, hedgehogs live in a variety of habitats where they dig their burrows, spend most of the daylight hours asleep, and emerge at night to forage. Hedgehogs can serve as reservoirs of *Salmonella* in many ways. During the night, villagers go to catch them as a meat source for the next day. During the rainy season, feces of animals including hedgehogs pollute the water sources such as rivers and wells. At the countryside many people are dependent on these sources for their potable water. In developed countries, people having exotic hedgehogs as pets have fallen sick with salmonellosis [10]. In these cases, the commonly detected *Salmonella* serotype has been *S*. Tilene [16]. Since we found several *S*. Tilene strains in our cattle and chicken meat samples during our previous study [13], we wanted to investigate a possible link between the *Salmonella* carriage of the production animals and hedgehogs, which share the same pastures for foraging. Indeed, we found hedgehogs in Burkina Faso to carry many *Salmonella* serotypes common also in the production animals, but no *S*. Tilene was detected, not in feces of the studied hedgehogs or of the other animals.

*S*. Muenster isolates were obtained from the feces of all the studied animal species and humans and their genetic relatedness in PFGE analysis was 90 to 95%. Thus, it is possible that the same strains of *S*. Muenster are able to infect many different hosts. Hedgehog feces might infect both cattle and swine foraging freely, since *Salmonella* can persist in the environment for several months to more than a year [41,42]. The production animals and the hedgehogs might all be able to transfer *Salmonella* further to the humans. We have previously shown the production animals to be potential carriers of virulent *Escherichia coli* to humans as well [43]. There is no previous information on the frequency of wild animals carrying enteropathogenic bacteria in Burkina Faso, apart from the *Salmonella* carriage of hedgehogs reported here.

## Conclusions

Our study revealed that both production and some wild animals commonly carry *Salmonella* in Burkina Faso. Some of the isolated *Salmonella* strains were genetically related to the human *Salmonella* strains and resistant to the common antimicrobials. As the humans and animals often live in close vicinity in Africa and the hygiene control of the meat retail chain is defective, high carriage rates of *Salmonella* and other potential pathogens of asymptomatic production animals can pose a major public health problem in Burkina Faso. Therefore, systematic surveillance of the infection sources and routes of the bacterial pathogens especially in the food production chain is needed to target the control actions to the critical points in the spread of the pathogens to the consumers.

## Methods

### Sampling

From 9 March to 25 August 2010, we collected 704 fecal samples from cattle (n = 304) and swine (n = 50) after slaughter at the central abattoir, and from chickens (n = 350) from the local poultry meat sellers in Ouagadougou, Burkina Faso, as previously described [43]. Hedgehogs (n = 25) were obtained from different villages across the country. Immediately after the animals were slaughtered, the fecal material was taken aseptically from the large intestine, 1 to 1.5 cm from the rectum. The samples were transported to the laboratory and kept at 4°C until the microbiological examination was started within 8 hours.

### *Salmonella* isolation and phenotyping

From each fecal sample, 25 g was enriched in 225 ml of buffered peptone water (Liofilchem, Teramo, Italy) at 37°C for 24 h. After that, 0.1 ml of an enriched sample was transferred into 10 ml of Rappaport-Vassiliadis broth and incubated at 42°C for additional 24 h before plating a loopful on Xylose Lysine Deoxycholate (XLD) agar (Oxoid, Basingstoke, England). Identity of the colonies with black center were confirmed biochemically using lysine and triple sugar iron agars and with API 20E (Biomerieux, Marcy l’Etoile, France). *Salmonella* isolates were serotyped with the somatic O and flagellar H anti-sera according to the Kauffman-White scheme [44]. Isolates of serotypes Typhimurium (including var. Copenhagen) were further phage typed [45].

### Antimicrobial susceptibility testing

Antimicrobial susceptibility of the isolates was tested by a standard disk diffusion method, and *Escherichia coli* RHE 6715 (ATCC 25922) was used for validating the antimicrobial test results [46]. The antimicrobial agents used were ampicillin (10 μg), chloramphenicol (30 μg), streptomycin (10 μg), sulphonamides(3 μg), trimethoprim (5 μg), tetracycline (30 μg), gentamicin (10 μg), nalidixic acid (30 μg), ciprofloxacin (5 μg), cefotaxime (30 μg), mecillinam (10 μg), imipenem (10 μg). Minimal inhibitory concentration (MIC) for ciprofloxacin (concentration ranging from 0,002 to 32 μg/ml) was determined by E-test (AB Biodisk, Solna Sweden) to the isolates resistant to nalidixic acid. MIC breakpoint ≤ 1 μg/ml was interpreted as susceptible [46].

### Genotyping

Isolates representing *Salmonella* serotypes, which were isolated from both the feces of the animals and from children in Burkina Faso, were subjected for genotypic analysis by PFGE. The serotypes included were Muenster (2 human, 7 cattle, 5 hedgehog, 3 swine and 3 poultry isolates), Typhimurium with antigen structure 4,5,12:i:1,2 (13 human and 4 poultry isolates) and Typhimurium var. Copenhagen with antigen structure 4,12:i:1,2 (3 cattle isolates), Virchow (2 human and 1 cattle isolates) and Ouakam (2 human and 1 swine isolates). In addition, four Albany isolates from two different animal species were included in the analysis (2 poultry and 2 cattle isolates). The 19 human *Salmonella* isolates were obtained from the National Public Health Laboratory in Ouagadougou, Burkina Faso and described in [17] and the 31 isolates of animal origin were from this study. For PFGE, the PulseNet protocol for *Salmonella* was used with the *Xba*I and *Bln*I restriction enzymes [47]. Briefly, agarose-embedded DNA was digested with 15 U of restriction enzyme (*Xba*I, Roche, Mannheim, Germany and *Bln*I, Fermentas International, Burlington, Ontario) at 37°C overnight. The restriction fragments were separated by electrophoresis in 0.5x TBE (HEPES for *S.* Ouakam) running buffer at 14°C for 20 h using the CHEF Mapper electrophoresis system (Bio-Rad Laboratories, Hercules, California, USA) with pulse times of 2 to 63 s, 120° angle, and 6.0 V/cm gradient. The agarose gels were stained with ethidium bromide, and the DNA banding patterns were analyzed by BioNumerics 5.10 software. *Salmonella* Braenderup H9812 was used as a standard. The bands within a size range from 33 kb to 1,135 kb were included in the analysis, and isolates differing even in one banding position were assigned as a new PFGE type. The dendrograms showing the grouping of the PFGE patterns were generated in BioNumerics using the composite of the patterns and the unweighted pair group method with arithmetic mean algorithm (UPGMA) with dice-predicted similarity value, 1% band optimization and 0,7% tolerance.

### Ethical considerations

Permission to conduct this study was obtained from the slaughterhouse authorities and the study protocol was approved by the Ethical Committee of Burkina Faso.

## Competing interests

The authors declare that they have no competing interests.

## Authors’ contributions

AK carried out the sampling and strain characterization and drafted the manuscript, TL and LA participated in the PFGE analysis, AST and NB supervised the sampling and strain isolation, AS and KH supervised the strain characterization and participated in writing the manuscript. All authors read, commented on and approved of the final manuscript.
